# Apparent Kinetics for Direct Oxidation of Iron Particles Determined from Tests at High Temperature in a Flat Flame Reactor

**DOI:** 10.1021/acs.energyfuels.6c00337

**Published:** 2026-03-23

**Authors:** Santiago Jiménez, M. Carmen Mayoral, Luis M. Romeo

**Affiliations:** † 120031 Instituto de Carboquímica-CSIC, Miguel Luesma 4, Zaragoza 50018, Spain; ‡ 16765 Aragon Institute of Engineering Research (I3A), Universidad de Zaragoza, Department of Mechanical Engineering, María de Luna 1, Zaragoza 50018, Spain

## Abstract

The results from experiments with 63–75 μm iron particles burning in high temperature gases in a flat flame reactor with 4–16% O_2_ are presented and compared with calculations, with the main goal of determining the vicinity of the measured oxidation rates to the external diffusion limit. The simulations consider oxygen diffusion through the particle’s boundary layer and encompass internal processes (diffusion, liquid convection, reaction, etc.) in the form of apparent kinetics in the outer surface of the particle. The main magnitudes monitored experimentally are particle temperature and especially its oxidation degree, based on the mass of oxygen absorbed; both are determined at different heights along the reactor, which results in detailed profiles with distance traveled or residence time. The assumption of combustion in the (external) diffusion limit notably overestimates the oxidation rate and the peak temperature of the particles. On the contrary, the introduction of different, progressively slower apparent kinetics for each oxidation stage, with Fe–O ratios corresponding to Fe → FeO → Fe_3_O_4_ → Fe_2_O_3_, results in an excellent agreement to the experimental data, particularly regarding the oxidation curves. The same kinetics are shown to fit equally well previous results obtained in tests with 75–90 μm, recently published by the authors.

## Graphical abstract

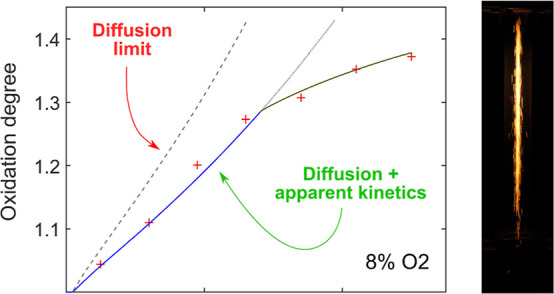

## 1 Introduction

The combination of iron oxide reduction and subsequent direct oxidation has been proposed as an efficient way of storing energy in large amounts, which in combination with a renewable source for the primary input may contribute to the necessary transition to a CO_2_-free energy generation pool.^1^ In recent years, and following this proposal, a number of works have focused on different aspects of iron combustion. In the last years, the experimental results of Ning et al. with laser-ignited particles in cold atmospheres,^2,3^ Ning et al. with particles in a flat flame reactor^4^ and Panahi et al. in a drop tube furnace^5^ have provided not only insights in the behavior of those particles during combustion, but also a reference with which the predictions of relatively simple models could be compared. In these works, the basic outcome was the profiles of temperature vs time in different conditions (namely O_2_ concentration), and the duration of solid/liquid oxidation derived from them. Fujinawa et al.^6^ extended a model for solid oxidation previously developed by Mi et al.^7^ for particle ignition to include also the liquid oxidation (which covers most of the complete process) from Fe to FeO, essentially transferring the limitation for the oxidation rate to the diffusion through the boundary layer. They found the calculated rates to be faster and the peak temperatures higher than the experimental ones in Ning et al.^2^ and Panahi et al.,^5^ and that in order to fit them adequately some internal diffusion of oxygen had to be considered; in the end, they used a different set of parameters for each experiment considered. There is, in the authors’ understanding, some uncertainty regarding the data of Ning et al.^2^ used by Fujinawa et al.,^6^ i.e. those corresponding to low temperature air around the particle. Fujinawa et al. seem to have taken a “representative temperature evolution” (Figure 11 in^2^) with a peak temperature 200–300 K below those later reported by Thijs et al.^8^ in the comparison of their calculations with Ning et al. experiments. In fact, the predictions of Fujinawa et al. given by the external diffusion limit would fit the data in^8^ without the need for internal limitations, at least in terms of maximal temperature reached.

As Thijs et al.,^8^ Ning and co-workers in recent works have assumed limitation by oxygen diffusion in the boundary layer during liquid iron oxidation and found good fits of the burn times reported by Thijs et al.^8^ and Ning et al.^3^ for iron particles ignited by a laser in a cold carrier gas.^3,9^ The corresponding particle peak temperatures, on the other hand, were either overpredicted in the low [O_2_] range or underpredicted above ∼25% O_2_;^8,9^ metal vaporization has been proposed to explain the latter deviation.^8^ Nguyen et al.^10^ considered internal diffusion in the form of apparent kinetics or ion diffusion through outer solid oxide layers (based on^7^), with reasonable fits of the scarce experimental data available regarding burn times and ignition temperature of isolated particles in the initial solid oxidation phase;^4,5^ in their calculations, however, this internal diffusion was irrelevant in the oxidation of the molten particle. Similar models were implemented by Mich et al.^11^ in order to investigate the flame front speeds of a population of particles; they again concluded that external diffusion may determine the oxidation rate except in the initial, solid stage, of minor relevance in terms of duration and heat release. Vance et al.^12^ have implemented apparent kinetics in their studies of flame propagation in iron clusters, too; they set the kinetics so that the ignition temperature was fixed at a certain value, and in practice external diffusion again limited oxidation afterward. Thijs et al.,^13^ in a very recent paper about the transport of oxygen outside and inside a burning iron particle, concluded that external diffusion is likely not the only limiting factor for oxidation after comparison with the experimental data already cited in this paragraph.^5,8^

In a recent, purely experimental work,^14^ the authors presented the results of a novel approach to the study of iron particle combustion at high temperatures, providing detailed information on the evolution of mass composition and internal structure of the particles, as well as their temperature profiles along a reactor in different oxygen concentrations (0.5–16% O_2_). A clear gradation of the oxidation rate and the particle temperature with [O_2_] was found in those tests, which also gave evidence for the existence of successive stages in the samples collected along the oxidation process, namely Fe → FeO → Fe_3_O_4_ → Fe_2_O_3_, with no overlapping between them. In the step Fe → FeO, two clear phases were observed in cross sections of the particles, with a receding iron core surrounded by iron oxide, whereas the progressive appearance of Fe_3_O_4_ and Fe_2_O_3_ in the samples could only be determined by X-ray diffraction, without physical separation inside the particles. The main novelty in the present work is the quantitative analysis of those oxidation rates, determined from the evolution of the O/Fe mass ratio in the samples collected at increasing residence times, by means of an intendedly simple model which considers external diffusion and apparent kinetics (i.e., simulating all internal processes as a reaction in the outer surface). With this model it is thus possible to establish the relevance of both terms or, in other words, the proximity to the diffusion limit in each step of the oxidation of the particles. In search of an extended range of oxidation degrees, especially at low concentrations, the tests reported in^14^ have been repeated with a slightly smaller size cut, 63–75 μm instead of 75–90 μm, although the results with the latter will also be compared with the simulations. The structure of this article is the following: Sections 2 and 3 briefly describe the experimental methods and summarizes the results obtained in the tests, Section 4 presents the model used in the calculations and Section 5 compares them with the experimental results.

## 2 Experimental Methods

The experimental setup and methods are identical to those described in detail in recent work,^14^ and for this reason they will only be briefly depicted here. Figure 1 shows a scheme of the reactor and the sampling probe used in these tests. The reactor consists of a down-fired flat flame burner connected to a 49 cm long quartz tube (inner diameter 60 mm). The burner’s sintered plate is water-cooled to avoid its excessive heating. The oxygen concentration in the burner’s flue gases, i.e. the uniform combustion atmosphere for the iron particles, was fixed by three mass flow controllers upstream (CH_4_, air and alternatively N_2_ and O_2_); the same conditions as in^14^ were explored: 0.5, 4.1, 8, 12 and 16% O_2_ (the full gas composition is given below in Section 4). The total flow was 2200 Nl/h (normal liters per hour). The iron powder was supplied by Pometon SpA as Ferblast MT-106; the analysis of this particular batch by the company indicated an oxygen content of 0.75%.^15^ It was thoroughly sieved in the range 63–75 μm. This fuel was fed at a rate of ∼50 g/h from a hopper equipped with a vibrating orifice (0.45 mm in diameter for this fuel and size cut). The fuel particles are gravity-driven through the burner into the combustion chamber along a metal tube (outer diameter 3 mm) together with a tiny flow of N_2_ (1 Nl/h) to avoid re-entrance of flue gases and thus condensation/clogging. Samples were collected at different heights below the burner (i.e., residence times) with probe equipped with a sintered bronze filter. The sampled gas is diluted with N_2_ at the probe’s tip to provoke a fast quenching. The probe itself is water-cooled and insulated. These samples were then analyzed by several techniques: microphotography (×40) to derive the particle size distribution; scanning electron microscopy (SEM) for the study of external and internal morphology; X-ray to determine their crystalline composition diffraction (XRD, Rietveld phase quantification); and thermogravimetric analysis (TGA) in H_2_ to obtain the ratio Fe/O in each sample. The uncertainty in this last technique is at most ±0.5% in mass loss in H_2_, which translates into a similar percentage in terms of oxidation degree, defined below.

**Figure 1:**
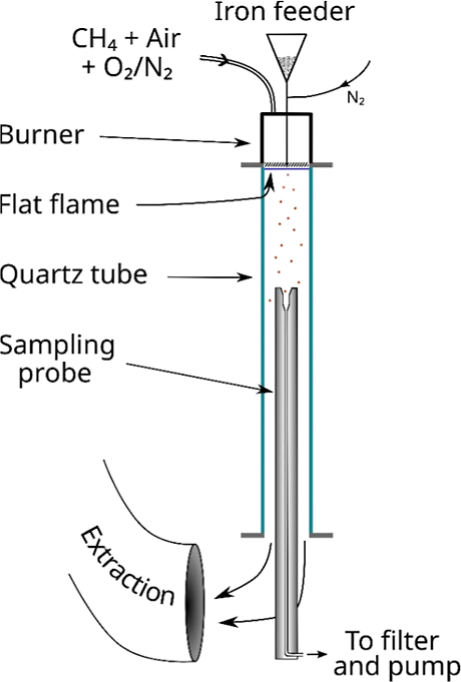
Schematic view of the flat flame reactor and the sampling probe used in the experiments.

The particle velocity and temperature profiles were determined in situ by optical means. The former, from the length of the particle traces in pictures taken with a DSLR camera. The particle temperature was measured with a 2D, 2-color pyrometer developed earlier and described in detail elsewhere.^16^ The use of two wavelengths (here, 640 and 830 nm) drastically reduces the uncertainty associated with the absolute value of the emissivity of the particles, as long as the gray-body assumption is applicable, for which some support is available in the literature.^17–19^ An attempt has been made to calibrate the pyrometer with iron inside the quartz tube, briefly summarized hereafter. Fine iron wires (30 and 50 μm, >99.5% Fe, Goodfellow) were set parallel to a 75 μm Pt wire in a probe, and finally placed in crossflow at different heights inside the quartz tube with 4.1% O_2_. The iron wire rapidly corroded, resulting in a much thicker wire (for instance, ∼112 μm oxide instead of the original 50 μm of pure iron) with a polycrystalline surface. Figure SI1 in the Supporting Information shows the aspect of the Pt and the originally 50 μm Fe wires after a series of tests. The signal in both wires in the pairs of pictures taken with the pyrometer were recorded and analyzed in this way: the Pt wire temperature was directly obtained from the intensity ratio in the wire images with the calibration curve established with a S-type thermocouple (as explained in^16^); the gas temperature at the probe placement was calculated based on the balance of heat convection and radiation;^20^ this gas temperature served in turn to estimate the temperature of the iron oxide wire, so in the end a correlation between signal ratio in the pyrometer (S1/S2, “1” for the 640 nm intensity) and iron temperature was obtained. The precision of this procedure depends largely on the uncertainty involved in the global emissivities of the materials, key in the calculation of the temperature gaps between gas and solid. That of platinum is small, but on the contrary iron oxide emissivity is high (∼0.7 according to^17,18^ ), and the highly altered surface shown in Figure SM1 for the corroded wire strongly suggests an even higher value; this results in large temperature gaps, above 300 K for a gas temperature ∼1900 K in spite of the relatively thin (original) iron wires used. Figure 2 shows the calibration curve of the pyrometer (S-type thermocouple data points + Planck’s theoretical fit) together with the new results with the iron wires; for the latter, two emissivities were considered, 0.7 and 0.9. In the authors’ opinion, it would be risky to derive sound conclusions from this graph regarding iron emissivity, except for the compatibility of the gray body assumption with the fit obtained. The (relatively) low temperatures attainable and the uncertainty in the determination of the iron oxide temperatures certainly privilege the use of the prior calibration of the pyrometer. Some further support for the correctness of the pyrometer’s calibration for iron oxide particles can be found in the particle temperature profiles obtained with the 4.1% O_2_ flame in this and the previous work:^14^ the morphology of the partially oxidized particles indicates that FeO is molten, whereas the remaining Fe cores stay mostly amorphous, pointing to a temperature between both melting points, 1650 and 1809 K; the peak temperature measured is 1700 K, consistent with those observations.

**Figure 2:**
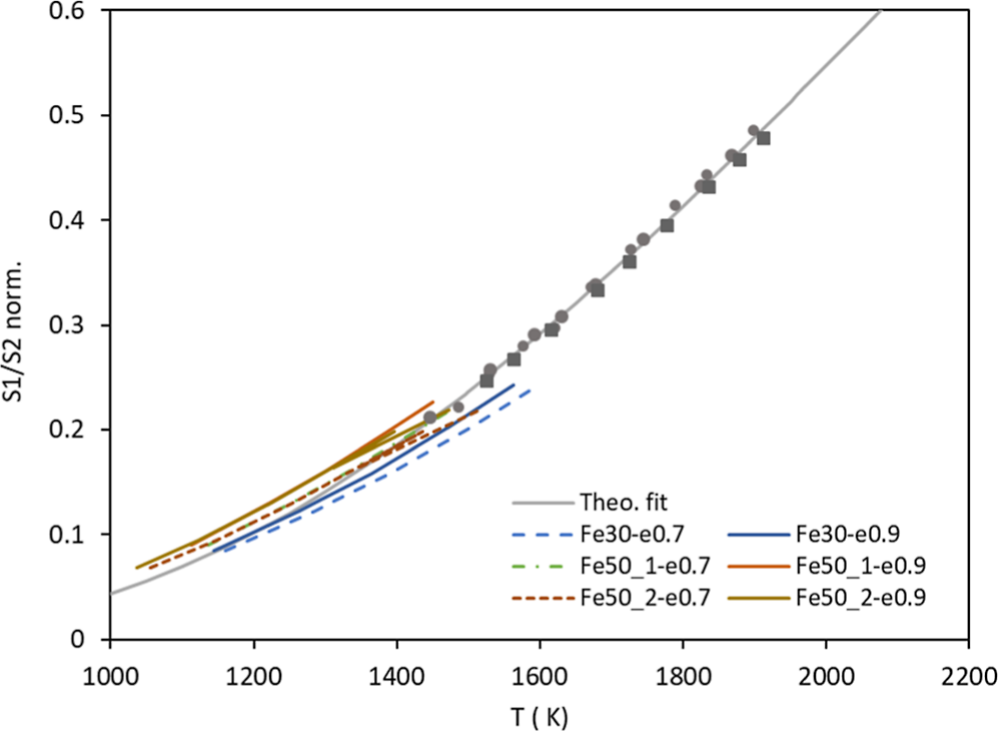
Calibration tests for the pyrometer. Dots: tests with 70 μm S-type thermocouple in situ. Gray line: theoretical fit to those results. Colored/shorter lines: tests with three iron wires, originally 30 and 50 μm in diameter; their temperature was estimated as explained in the text for two values of emissivity (0.7 and 0.9).

## 3 Experimental Results

The general aspect of the iron “flames” is shown in Figure SI2 in the Supporting Information. The particle traces along the reactor are similar to the ones previously published for the larger size cut, although slightly shorter for 12 and 16% O_2_ (in those conditions the traces end within the reactor, whereas for [O_2_] ≤ 8% they are visible all along it). The same can in fact be said regarding the particle temperature profiles presented in Figure 3. The peak temperatures are very similar for 63–75 μm and 75–90 μm, except for 8% O_2_, ∼40 K higher for the smaller particles. In all the cases this peak is reached earlier (faster) with this size cut. As explained in detail in,^14^ the procedure set to determine the temperature at a certain height is biased by the hottest particles; for this reason, the large uncertainty bars in the tail of the profiles of the highest concentrations are caused by a number of particles where combustion still proceeds, rather than by an average cooling of the ensemble of particles.

**Figure 3:**
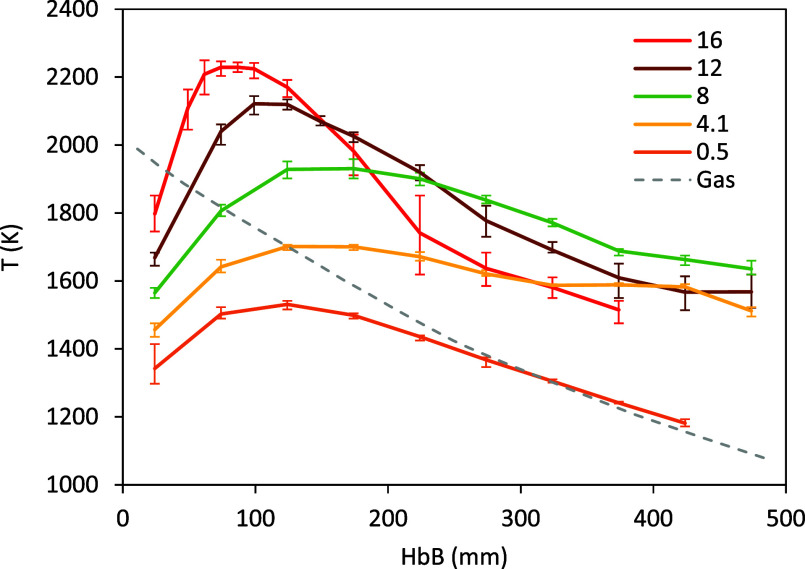
Particle temperature vs height below the burner (HbB) in the conditions explored in this work (0.5–16% O_2_, 63–75 μm Fe particles), together with an average gas temperature profile. The legend indicates [O_2_] in %.

The gas temperature profile in Figure 3 represents an average of the actual profiles measured for the different concentrations (±20 K around this average,^14^). Figure 4 shows the gas velocity based on this profile assuming plug flow, together with individual particle speeds. An average speed profile, also plotted in this figure, will be used in the calculations presented in Section 5. As expected, the 63–75 μm particles’ velocity is slightly lower than that of the 75–90 μm size cut, and their average residence time in the reactor ∼10% longer. Figure 5, which shows the oxidation degree vs distance traveled and [O_2_], is the main reason for extending the previous work to slightly smaller particles: there are more significant data in the upper end of the curves, and a clearer representation of the “ending” of the combustion curves. The oxidation degree in axis Y is defined as

1 $$OD:=\frac{m_{T}}{m_{Fe}}=\frac{m_{O}+m_{Fe}}{m_{Fe}}=1+\frac{m_{O}}{m_{Fe}}$$

where the ratio m_O_/m_Fe_ is determined by thermogravimetric analysis as explained in Section 2. The horizontal, dotted lines in Figure 5 mark the initial iron and the oxidation degrees corresponding to FeO, Fe_3_O_4_ and Fe_2_O_3_. The plot depicts roughly straight lines in the first oxidation step (up to FeO), followed by marked decelerations afterward. The evolution of the crystalline composition of the samples, shown in Figure 6, is the same presented in,^14^ i.e. Fe → FeO → Fe_3_O_4_ → Fe_2_O_3_, with no overlapping of the steps. The final fraction of Fe_2_O_3_ is higher for these particles than it was found for the coarser sizes (∼30% vs 5%). Figure SI3 (in the Supporting Information) presents the diffractograms of Figure 6 and the corresponding fits found by the Rietveld method.

**Figure 4:**
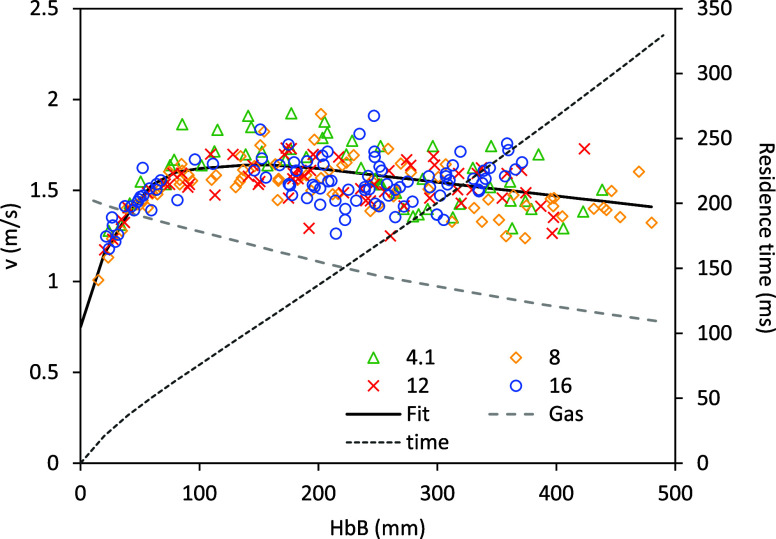
Measured particle velocities vs height below burner (HbB) in the explored conditions ([O_2_] in legend,%), as well as gas velocity calculated from the measured temperature profile. A fit for the particle velocity and, based on it, an average residence time vs HbB are also plotted.

**Figure 5:**
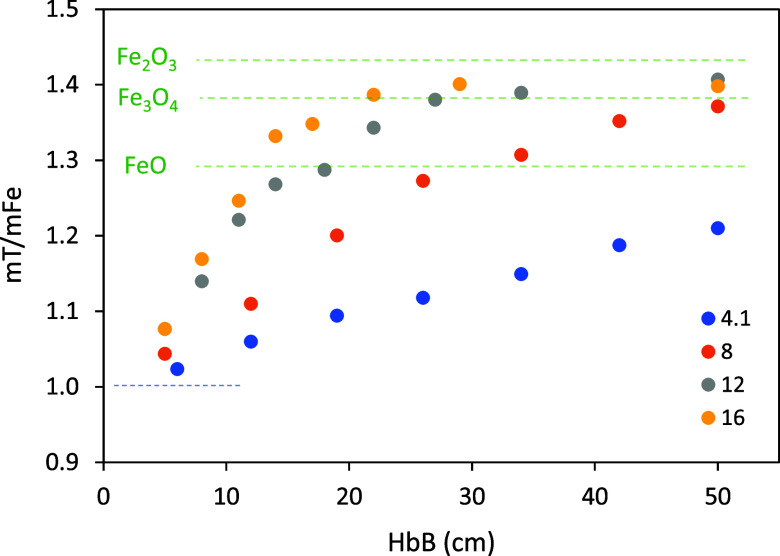
Oxidation degree vs distance traveled by the 63–75 μm iron particles in four oxygen concentrations (% in the legend).

**Figure 6:**
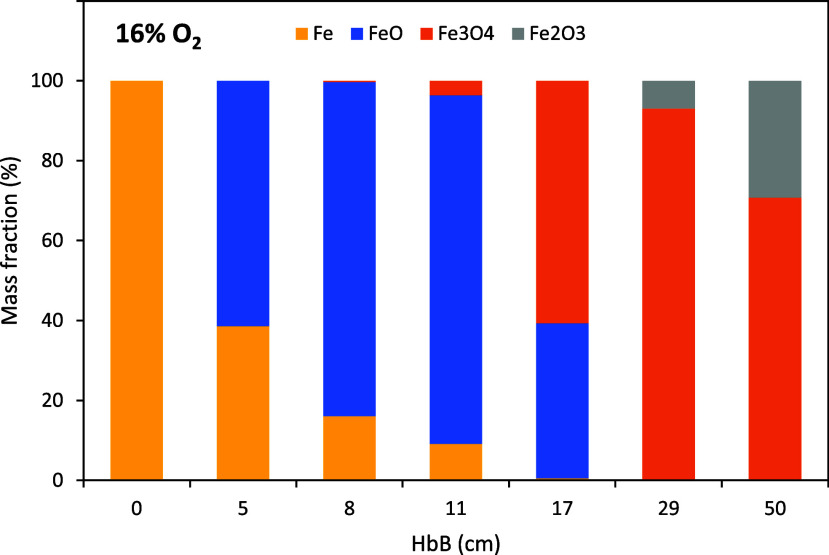
Evolution of the crystalline composition of the particles along the 16% O_2_ series, determined by XRD.

The SEM micrographs corresponding to the new size cut show a pattern very similar to the one reported for 75–90 μm and are omitted here for the sake of brevity. For the same reason, only the evolution of the particle size distribution in 12% O_2_ is shown in Figure 7, with the rest available as Figure SI4 in the Supporting Information. The particles steadily grow along oxidation until the last stages, where some of them swell extraordinarily. This late loss of uniformity, already commented above regarding temperature or in the authors’ previous detailed study of the morphology based on series of SEM images, should make the reader keep in mind that in this uppermost oxidation range the curves in Figure 5 represent the average behavior of a population of particles, rather than the evolution of a single one. Particle breakup seems to occur a bit more frequently in this size cut than with the larger particles, but it is still of very little relevance in statistical terms. Figure 8 plots the average diameter in the main mode of each distribution vs the corresponding oxidation degree. As explained in,^14^ assuming a compact or dense particle is not in agreement with the observations of their internal structure, with significant voids especially after completing the oxidation to FeO, nor it does fit the evolution of the average diameter (the dot-dashed curve in Figure 8 is calculated under this assumption); instead, an empirical correlation will be used in the calculations shown later:

2 $$d_{p}=d_{p,0}+d_{p,0}\cdot \eta \cdot \left(OD-1\right)$$

where η is determined by best-fit to the experimental data. According to Figure 8, η = 1.1 for the 63–75 μm cut. A slightly smaller value, η = 1.05 was found for 75–90 μm particles.

**Figure 7:**
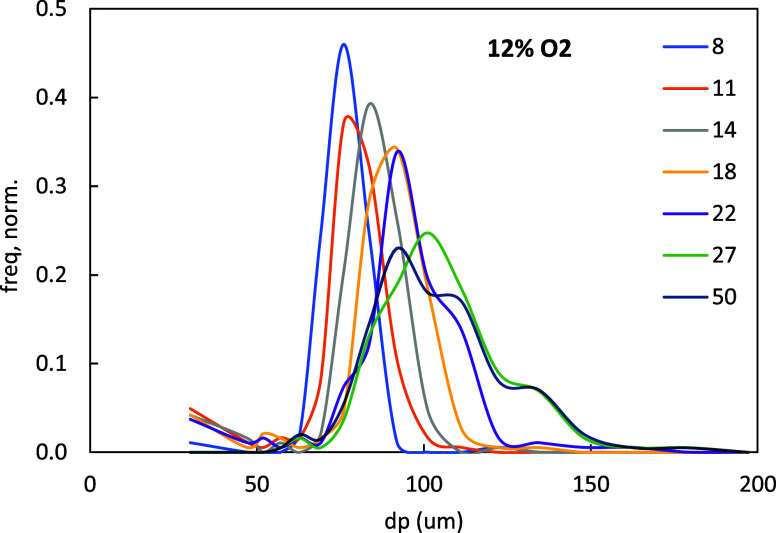
Particle size distributions (number-weighed) in the samples collected with 12% O_2_, 63–75 μm. Legend: HbB in cm.

**Figure 8:**
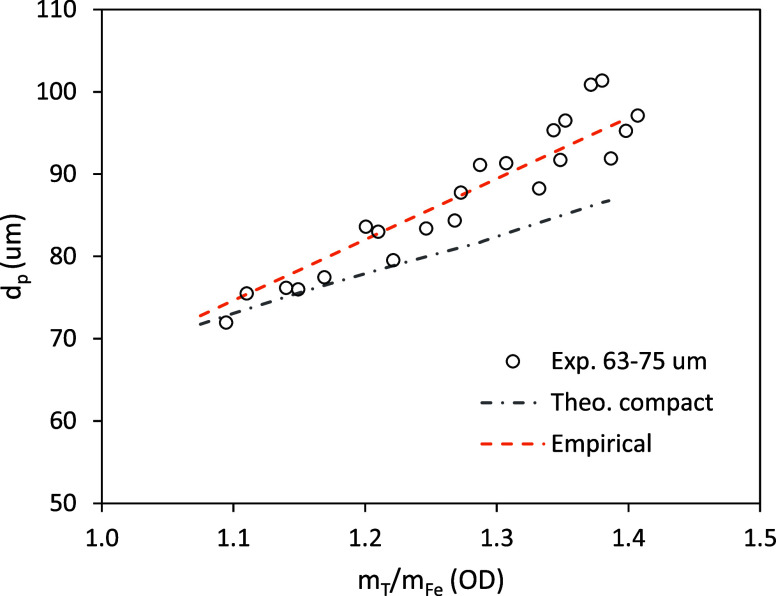
Average particle diameter (number-weighed) vs oxidation degree.

Finally, for the sake of completeness, Figure SI5 in the Supporting Information compares the fraction of Fe determined by TGA with the value derived from the XRD analysis. As it happened with the 75–90 μm particles, XRD overvalues the content of iron oxide in the range of low OD’s, whereas both analytical techniques coincide in more oxidized samples. This is attributed to the layered structure of the Fe–FeO particles and the limited penetration of X-rays. This might explain also a slightly smaller deviation for the 63–75 μm particles, but a detailed study is beyond the scope of this work.

## 4 Combustion Model and Parameters

As stated in the Introduction, the main goal in this work was to determine whether the oxidation of the particles proceeds at rates given by the so-called (external) diffusion limit or if, on the contrary, one or several internal limitations play a significant role. An approach based on apparent kinetics is convenient in this regard, since in this formulation internal diffusion of gas or ions, liquid convection and/or real kinetic limitations are represented by a reaction in the outer surface of the particle or molten droplet,^21^ with only two free parameters to be adjusted in each case.

Based on the flow rates of fuel and gas, iron particles are assumed to burn in isolation (the interparticle distances are above 100 particle diameters in average). They are also assumed to be thermally thin; any internal gradient is neglected. The particles exchange heat with their surroundings by convection and radiation

3 $${\mathrm{Q̇}}_{conv}=Nu\cdot \pi \cdot d_{p}\cdot \lambda_{g}\cdot \left(T_{\infty }-T_{p}\right)$$

4 $${\mathrm{Q̇}}_{rad}=-\pi \cdot {d_{p}}^{2}\cdot \varepsilon \cdot \sigma \cdot \left({T_{p}}^{4}-{T_{w}}^{4}\right)$$

where the subscripts *p*, ∞ and *w* refer to the particle, the bulk gas and the surroundings in radiative terms, respectively; λ_*g*_ is the conductivity of the gas; *Nu* is the Nusselt number, ε the emissivity of the particle and σ the Stefan–Boltzmann constant.

The contribution of species other than O_2_ to the oxidation of the particles is neglected. As shown below, the concentration of H_2_O is similar in all the atmospheres explored. No reaction was observed in the absence of oxygen (profile for 0.5% O_2_ in Figure 3 and an analysis of a sample with long residence time in this condition in^14^), which supports this assumption. The rate at which oxygen is “captured” by the particle is given by the equality of oxygen diffused through the boundary layer and the one that reacts with the solid or liquid fuel. For the latter, as just explained, apparent kinetics is used here

5 $${ṁ}_{O2}=Sh\cdot \pi \cdot d_{p}\cdot \rho_{g}\cdot D_{O_{2}}\cdot \left(Y_{O_{2},\infty }-Y_{O_{2},S}\right)$$

6 $${ṁ}_{O2}=\pi \cdot {d_{p}}^{2}\cdot A_{a,i}\cdot e^{-E_{a,i}/R\cdot T_{p}}\cdot P_{g}\cdot \frac{W_{g}}{W_{O_{2}}}\cdot Y_{O_{2},S}$$

*S* refers to the external surface of the particle. *Sh* is the Sherwood number, ρ the gas density, *D*_O_2__ the diffusivity of oxygen, *Y*_O_2__ its mass fraction and *W* the molar weight. *A*_*a*,*i*_ and *E*_*a*,*i*_ are the pre-exponential factor and the activation energy defining the apparent kinetics, where *i* refers to the three consecutive oxidation stages observed in the samples collected, namely Fe → FeO, FeO → Fe_3_O_4_ and Fe_3_O_4_ → Fe_2_O_3_.

The Fe–O phase diagrams, which at high temperatures are largely the result of theoretical extrapolations from metallurgical studies (e.g.,^22^), predict the existence of a generic (L2) liquid oxide, in coexistence with liquid iron (L1) only until O ≈ Fe in a molar base (which agrees well with the results presented in^14^ regarding the first “step” in the oxidation, Fe → FeO). The upper end for the L2-alone region depends on the extrapolation: Muller et al.^23^ would admit ratios corresponding to pure Fe_2_O_3_, whereas others (e.g.,^6,22^ ) set the limit at a ratio equivalent to roughly 30% Fe_2_O_3_-70% Fe_3_O_4_, which just covers the maximal value found in these tests. In the cooling, the diagrams suggest a transformation to Fe_3_O_4_(*s*) with degasification if extra O was present in the liquid; the presence of Fe_2_O_3_ in the samples would correspond, in this scheme, to a later oxidation in solid state. The formation of large cavities in fast oxidations of iron bars and particles has been attributed in the past to that potential degasification.^23,24^ Some support for an oxidation post-solidification can be found in the intensity profiles of individual particles shown by Ning et al.^25^ and Panahi et al.;^5^ the corresponding particle compositions were not determined, though.

Apart from the aforementioned differences in the extrapolations, there is, in the authors’ opinion, very little evidence on the applicability of these metallurgical phase diagrams to the extremely fast processes involved in the combustion of pulverized iron; Tóth et al.^26^ previously expressed similar concerns. It is unclear, for instance, where to “stop” the traces along the particle cooling across the diagrams: they indicate that only Fe, Fe_3_O_4_ and Fe_2_O_3_ are stable at room temperature, whereas in many samples collected in the reactor FeO is the most abundant species (it was also found in relevant fractions in the products of a self-sustained iron burner^27^). In this work, it will be assumed that the Fe–O ratios measured in the samples at room temperature are equal to the ratios in the particles and droplets sampled at high temperature. The experiments presented in^14^ and more briefly in this article have provided evidence for the existence of at least two stages: coexistence of a Fe core with a FeO shell, and beyond pure FeO; this may justify a potential change in the oxygen absorption rate, i.e. different apparent kinetics here.

The heat generated in the oxidation is proportional to the oxygen absorbed, and depends on the reaction considered in each range of Fe–O ratios, represented by the species found in the solid samples

7 $${\mathrm{Q̇}}_{chem}={ṁ}_{O2}\cdot \Delta H_{i}$$

The corresponding reaction enthalpies are

$$\begin{array}{l} {\Delta H}_{1}\left(Fe\to FeO\right)=-544\;kJ/{mol\;O}_{2}\\ {\Delta H}_{2}\left(FeO\to {Fe}_{3}O_{4}\right)=-642\;kJ/{mol\;O}_{2}\\ {\Delta H}_{3}\left({Fe}_{3}O_{4}\to {Fe}_{2}O_{3}\right)=-472\;kJ/{mol\;O}_{2} \end{array}$$

The first step (Fe → FeO) represents the largest fraction of the oxygen incorporated (Figure 5), and fully covers the region of highest temperatures. The uncertainties associated with the formation of Fe_2_O_3_ (or, in other words, to whether those Fe–O ratios are reached in liquid state or after solidification) are of very minor relevance in what regards the oxygen captured and the particle temperature profile due to the small amounts of oxygen involved in that process (Figure 5) and, consequently, the little energy released (with no noticeable effect in Figure 3).

The Stefan flow has been neglected in eq 5. Accounting for it implies substituting the last parentheses in that equation, (*Y*_O_2_,∞_-*Y*_O_2_,*S*_) with

8 $$\ln\left(1+B_{M}\right),where{\;B}_{M}=\frac{Y_{O_{2},\infty }-Y_{O_{2},S}}{Y_{O_{2},S}-1}$$

This would force a numerical solution for eqs 5 and 6, plus an iterative calculation around *T*_*p*_, which is undesired especially if the model is to be implemented in more complex CFD simulations of industrial facilities. The underestimation of the oxygen mass flow rate if the Stefan flow is dismissed is, for 16% O_2_ and in the diffusion limit (i.e., the worst case within the conditions explored in this work), below 9%. This value decreases with oxygen concentration and/or when apparent kinetics play a relevant role, as commented later.

The evolution of particle temperature is calculated through

9 $$\frac{d\left(c_{p,p}\cdot m_{p}\cdot T_{p}\right)}{dt}={\mathrm{Q̇}}_{conv}+{\mathrm{Q̇}}_{rad}+{\mathrm{Q̇}}_{chem}$$

where the effect of iron evaporation has been neglected based on the calculations of Thijs et al.^8^ and Fujinawa et al.^6^ and the relatively low oxygen concentrations used in the experiments.

The Nusselt and Sherwood numbers are calculated with the Ranz–Marshall correlations, based on the particle diameter and the average slip velocity determined experimentally (Figure 4)­

10 $$Nu=2+0.6\cdot Re^{1/2}\cdot Pr^{1/3}$$

11 $$Sh=2+0.6\cdot Re^{1/2}\cdot Sc^{1/3}$$

All the gas properties are evaluated with Cantera’s package for Matlab^28^ at a “film” temperature $T_{f}=\frac{1}{2}\cdot \left(T_{p}+T_{\infty }\right)$ . The thermodynamic data for all species in the gas and the particles are taken from the NIST-JANAF series.^29^

A 0-D, pseudostationary calculation reproduces the process observed in the experiments, from the injection of dry, pure iron particles at room temperature. The gas and (average) particle velocity profiles are taken from Figure 4. The molar composition of the bulk gas in each condition explored is presented in Table 1.

**Table 1:** Gas Composition Downstream of the Flat Flame (molar Percentage)

flame [O_2_]	CO_2_	H_2_O	N_2_	O_2_
0.5	7.64	15.28	76.59	0.49
4.1	7.66	15.33	72.95	4.06
8	8.23	16.46	67.31	8.00
12	8.81	17.62	61.57	12.00
16	9.13	18.25	56.62	16.00

The quartz wall temperature profile (here, 750 → 450 K, linear decay) plays a very minor role in the radiative term of eq 9: as shown later, convection largely dominates the initial particle heating, and the actual wall temperature is irrelevant once the particle is ignited. Emissivity is set equal to 0.7.^17,18^ Ignition is supposed to occur at 1000 K. The large uncertainty in this parameter ( ^7,12^ and references therein) has, again, a very minor effect in the computed oxidation curves, due to the short time involved in the heating to that temperature, compared to the actual oxidation in the conditions explored. Only Fe melting is considered, at 1809 K, without it being an interruption for oxidation.

The authors are aware of the potential relevance of considering a number of size classes even when working with sieved cuts in order to reproduce the curvature of the OD vs t results (as for coal in^30^). Nevertheless, a single particle diameter is considered in this work as representative of the distribution used in the tests for these reasons:

- First, because the “curves” shown in Figure 5 are not actually very curved. They rather present a marked change in slope after a long linear segment, which is not what one would expect if a significantly wide distribution were used.
- Then, due to the narrowness of the actual distributions measured in micrographs (Figures 7 and SM3), and the duality solid–liquid of the fuel. This latter aspect actually affects the way in which the distribution should be determined. Figure SI6 (in the Supporting Information) presents the distribution of the 63–75 μm cut of the fuel as measured with a laser diffractometer (Malvern Mastersizer S), together with the distribution derived from manual inspection of a sample with (partially) molten particles and low OD (4.1% O_2_, 19 cm). Although the mean diameter, once both distributions expressed as number-weighed, is roughly the same, the former distribution is much broader. The reason for this is most likely the amorphous nature of the raw fuel, in contrast with the spherical shapes encountered in the partially burnt samples. Both can be compared in Figure SI7, where the great uniformity of the particles is apparent as soon as they are molten.
- Finally, because this being the first attempt, to the authors’ knowledge, to fit iron oxidation curves in a mass basis, a single diameter allows for clearer conclusions, once the previous conditions are met.

Based on Figure 8, a diameter of 72 μm is taken as average in mass, since the main experimental results with which the simulations are to be compared (Figure 5) are mass-weighed. The size distributions in the least oxidized samples are so narrow that the changes from number-weighed to mass- or volume-weighed mean diameter are small (within 2–3 μm). The same argument leads to a particle of 82 μm as representative of the 75–90 μm size cut.

## 5 Comparison of Experimental Results and Simulations

### 5.1 Comparison with Previous Calculations

Figure 9 shows the peak temperature predicted by the model just described when applied to a 50 μm initially at high temperature in a varying oxygen concentration in gas at room temperature, compared to the calculations of Thijs et al.^8^ and Fujinawa et al.^6^ mentioned in the Introduction. In order to reproduce their conditions, only the oxidation of Fe to FeO limited by external diffusion (infinite apparent kinetics) was assumed. The good agreement shown in Figure 9 served as a first validation of the code.

**Figure 9:**
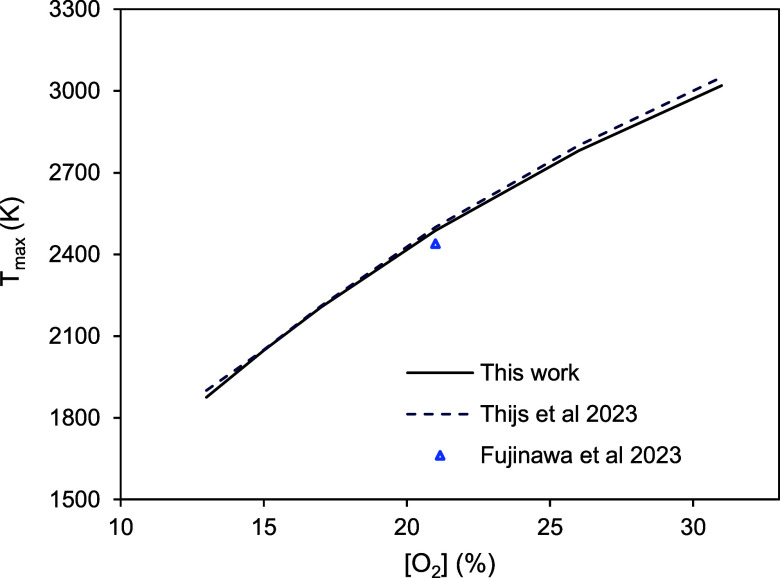
Maximum temperature predicted for a 50 μm particle ignited by laser in a room temperature, 21% O_2_ atmosphere, compared to the calculations of Thijs et al.^8^ and Fujinawa et al.^6^

### 5.2 Heating in an Inert Atmosphere

The particle temperature evolution in a nearly inert atmosphere (0.5% O_2_) was used as a validation test for the model and boundary conditions implemented to simulate the tests described in Section 2. Fitting this temperature profile is important to later evaluate the increase in maximal temperature due to oxidation. Figure 10 presents the comparison of measurements (Figure 3) and calculations for a 72 μm particle without oxidation (*Q̇*_chem_ = 0). The use of the “original” gas profile, measured in the absence of particles, results in a max temperature ∼200 K above the measured one. This was attributed to dismissing the cooling of the gas in the near-axis region close to the particle injection tube. Although the iron feeding rate (∼50 g/h) is negligible compared to the total gas flow rate (∼2600 g/h), initially the particles are confined in a smaller radius; if only the gas in a truncated cone with an upper radius of 6 mm, height 5 cm and lower radius 12 mm (which reproduces approximately the shape of the particles’ initial trajectories in the images with a few mm of outer margin) is assumed to exchange heat with the mass-flow of particles, the particle temperature profile perfectly fits the measured one. Figure 10 also shows the corresponding expected gas temperature profile along the reactor’s axis. In what follows, this “corrected gas profile’ is used for the simulation of a single particle in that flow. The effect of this correction, and the uncertainty it introduces in the calculation of the oxidation curves, will be further treated later in subsection [Sec sec5.4].

**Figure 10:**
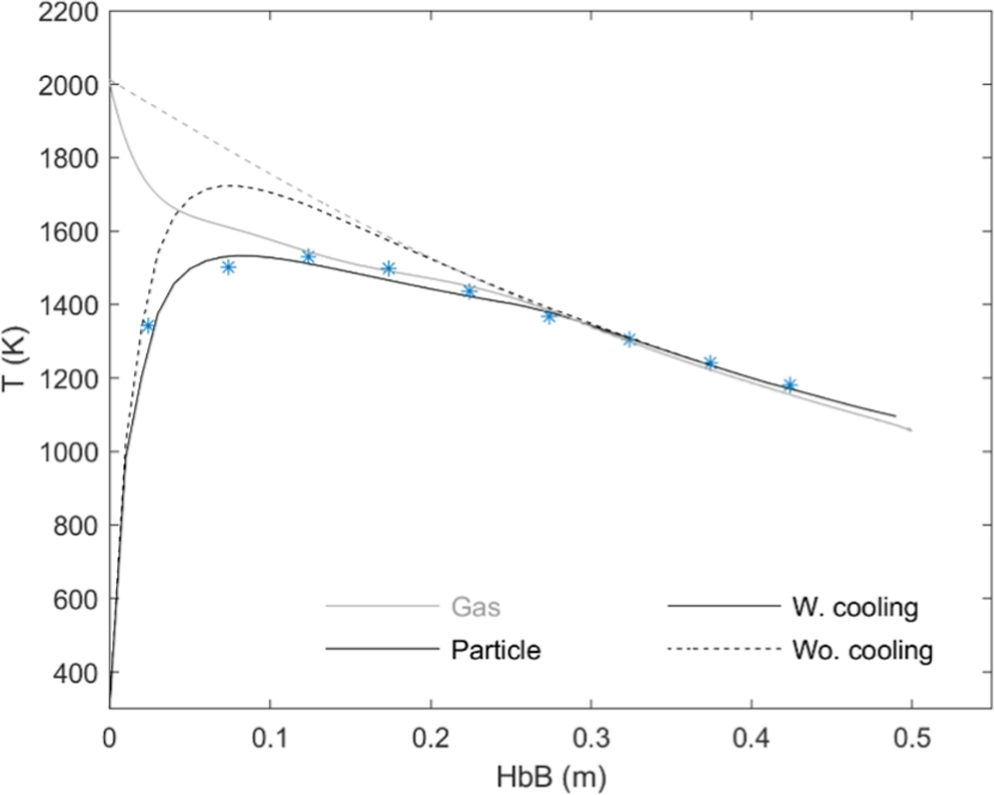
Comparison of particle temperature measurements and calculations in a nearly inert atmosphere (0.5% O_2_). Continuous lines: with initial gas cooling (see text); dashed lines: with the original gas temperature profile. Gray lines for gas.

### 5.3 Fitting of the Combustion Curves

Figure 11 compares simulations and tests regarding the evolution of the oxidation degree (i.e., the O–Fe ratio) in the conditions explored, while Figure 12 shows the equivalent graphs for particle temperature. It is evident in Figure 11 that the oxidation rate limited only by the diffusion of oxygen to the outer surface of the particle largely exceeds the actual rate in all conditions; also, in terms of particle temperature (Figure 12), with an overestimate of up to ∼700 K for 16% O_2_. Since, as explicitly shown below, convection plays a major role in heat loss by the burning particles, this overestimation was actually expected based on the calculations summarized in Figure 9 for iron particles burnt in room temperature atmospheres, with values already similar to those measured in the present work in high temperature gases. Fujinawa et al.^6^ found a similar deviation in the maximal temperature predicted at the external diffusion limit for tests with 21, 50 and 100% O_2_ in a drop-tube furnace, i.e. in high temperature gas (as well as one in room temperature gas attributed to Ning et al.,^2^ although in this case the data used is unclear, as commented in the Introduction). Those authors proposed a “heuristic model” which considered internal ion diffusion (as in Mi et al.^7^) with pre-exponential factors defined ad-hoc for each test and condition in order to fit those results.

**Figure 11:**
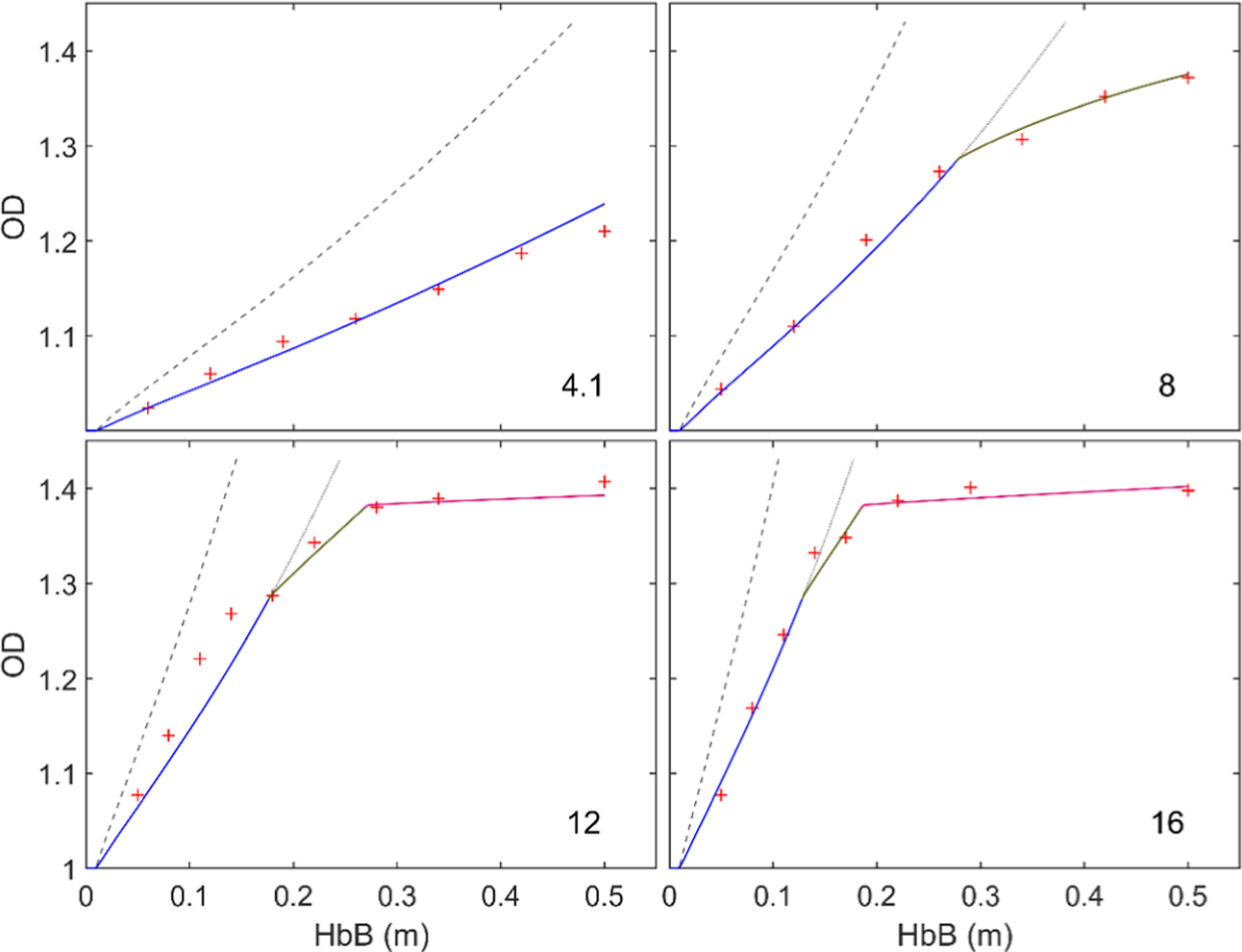
Comparison of experiments and simulations of oxidation degree vs distance traveled along the reactor. Dashed lines: predictions made for a 72 μm particle assuming limitation by external diffusion; continuous, blue → gray: with fixed kinetics (1 in Table 2); continuous, colored: with different kinetics for each oxidation reaction (1–3 in Table 2). The number in each graph indicates the oxygen concentration in %.

**Figure 12:**
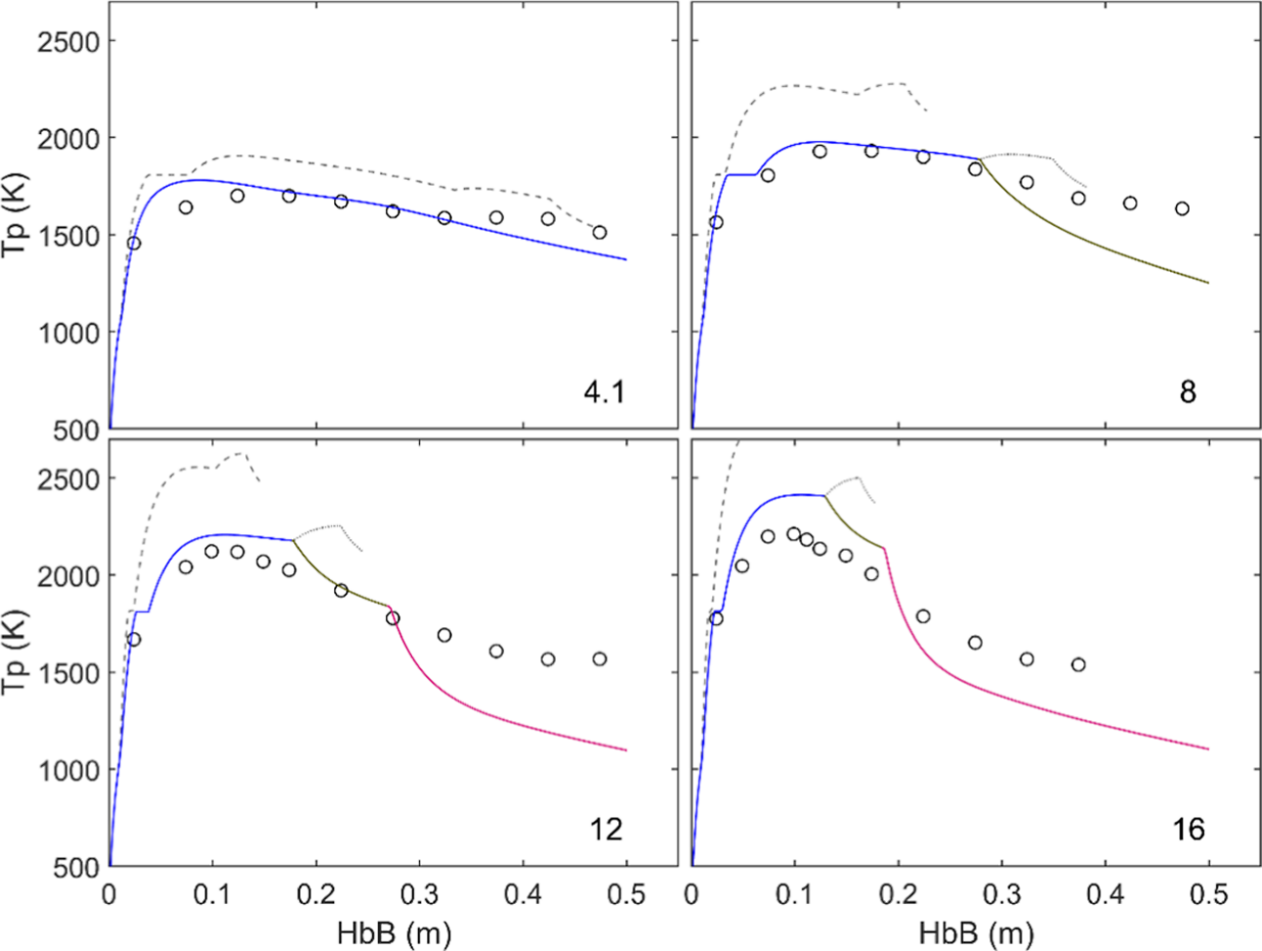
Measured and calculated particle temperature profiles. Lines as in Figure 11. The max. Temperature in 16% O_2_ with fixed kinetics (1 in Table 2) reaches 2900 K.

In the present work, the goal was to determine a set of apparent kinetics applicable to all the conditions explored in the tests. No attempt was made to relate the kinetics with a physical model for the diffusion of ions, liquid recirculation within the molten iron/iron oxide or oxidation reaction. The choice of first order kinetics in eq 6, for instance, was simply based on the simplicity of the analytical solution of eqs 5–6. In the light of the nearly constant factor between the rate predicted by external diffusion alone and the one observed in the plots of Figure 11, a relatively low activation energy was set and the pre-exponential factor was determined by best fit to the data. First, fixed kinetic parameters (“1/global” in Table 2) were set for all the reactions, which resulted in a very good fit of the oxidation curves in Figure 11 except for the range of OD > 1.3, where again the simulation overpredicts the oxidation rate. Greater restrictions were subsequently set for the second and third steps in the oxidation, as shown in Table 2. In the fitting of the former, some dependence on the particle temperature was observed, which explains the change in activation energy. In the latter oxidation stage (Fe–O ratios corresponding to Fe_3_O_4_ → Fe_2_O_3_), due to the little data available, simply *a* factor of 0.01 was added to the first kinetics.

**Table 2:** Apparent Kinetics for the Oxidation of Iron Particles

*i*	reaction	*E*_a,i_ (J/mol)	*A*_a,i_ (kg/m^2^sPa)
1/global	Fe → FeO	12,000	8 · 10^–5^
2	FeO → Fe_3_O_4_	40,000	1.2 · 10^–4^
3	Fe_3_O_4_ → Fe_2_O_3_	12,000	0.01·A_a,1_

When the three successive kinetics in Table 2 are implemented in the model, an excellent agreement of the simulations with the oxidation curves is found in Figure 11, which covers a wide range of conditions, from 4.1 to 16% O_2_. As highlighted in the authors’ previous work^14^ and also noted above, the results regarding the last stage of oxidation should not be seen as the description of every particle’s behavior (which is generally true for prior stages), but instead as an average of the evolution a very diverse population.

The corresponding fit of the temperature profiles in Figure 12 is reasonably good, with a progressive deviation in the maximal temperature predicted for the highest oxygen concentrations. This is attributed to the likely evaporation of iron at these temperatures, not accounted for in the model. The presence of a visible plume of very fine particles for [O_2_] ≥ 12%, briefly commented in^14^ and also observed in these tests with smaller iron particles, would be consistent with this attribution. Fujinawa et al.^6^ and Thijs et al.^8^ considered vaporization in their calculations, which pointed to an effect on the peak temperature only above ∼2500 K.

Finally, note that the aforementioned averaging “forced” by the model in the last stage of oxidation has a more complex interpretation in terms of particle temperature than it had in mass, especially when compared to the experimental data in the tail of the profiles, in which, as explained above, a number (not mass) of hot particles may result in a noticeable shift toward higher temperatures.

### 5.4 Discussion of Several Aspects of the Simulations

This subsection gets into some details of the simulations; in all the cases, the results correspond to the model with the three apparent kinetics shown in Table 2. Figure 13 illustrates the evolution of the different species in a particle along the reactor in 16% O_2_, normalized by its initial mass and under the assumption, explained in Section 4, that the Fe–O ratio is the same in the liquid than in the samples at room temperature. There is an excellent qualitative agreement of this plot with the XRD data shown in Figure 6; quantitatively, however, this technique tends to overestimate the ratio FeO/Fe most likely due to the limited penetration of the X-rays in the particles and their layered structure in that oxidation stage, as explained in^14^ and again commented above in this paper (Figure SM4).

**Figure 13:**
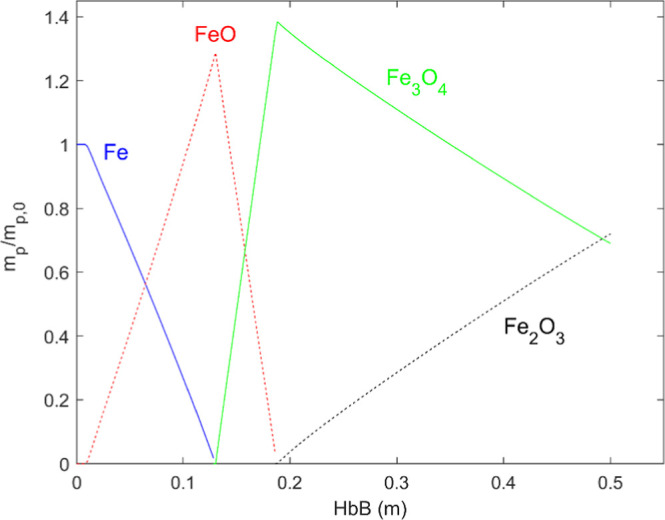
Normalized evolution of mass in a 72 μm particle; case of 16% O_2_.

Figure 14 plots the heat flows as a function of the residence time in the reactor, with 12% O_2_. The end of the two first oxidation stages are clearly marked by a drop in the heat generated (of course, only if separate kinetics are considered). Whereas steady states are identifiable in the steps leading to Fe_3_O_4_ and Fe_2_O_3_, the maximal iron oxidation rate and thus heat generation are found at the end of the first stage (Fe → FeO). In the conditions explored in these experiments, conduction/convection dominates over radiation in the heat loss. This reduces the relevance of the uncertainty in the emissivity of the particle; on the other hand, it highlights the importance of correctly modeling the parameters affecting heat convection. In this regard, Figure 15 shows the Nusselt number calculated for a 72 μm particle based on the velocity profiles of Figure 4 and correlation 10, which implies roughly a 10% increase with respect to the case of a stationary particle. As for the calculation of the external diffusion of oxygen, the error associated with neglecting the Stefan flow can be determined a posteriori with eq 8, once the oxygen partial pressure at the particle’s surface is known: even at 16% O_2_, the underestimation is below 5%. Note that this does not affect the quality of the curve fits in this amount: slightly different kinetics would have been obtained, with the numerical drawbacks commented above.

**Figure 14:**
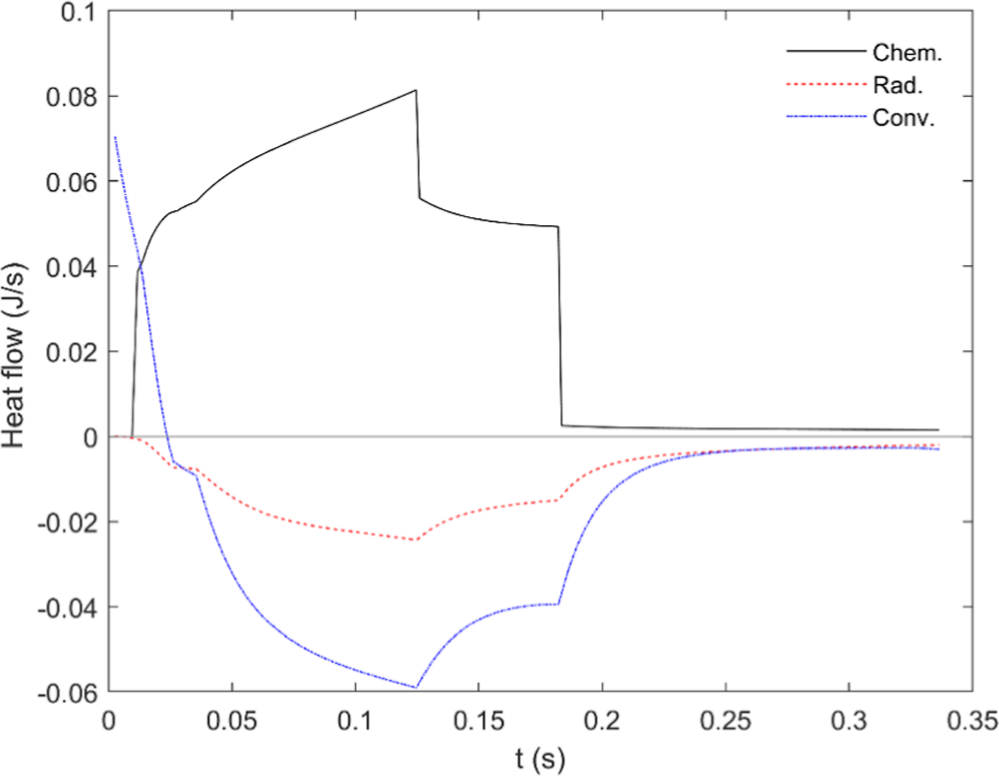
Heat flows vs residence time, case of 12% O_2_, 72 μm. Positive values correspond to heat generated or absorbed by the particle.

**Figure 15:**
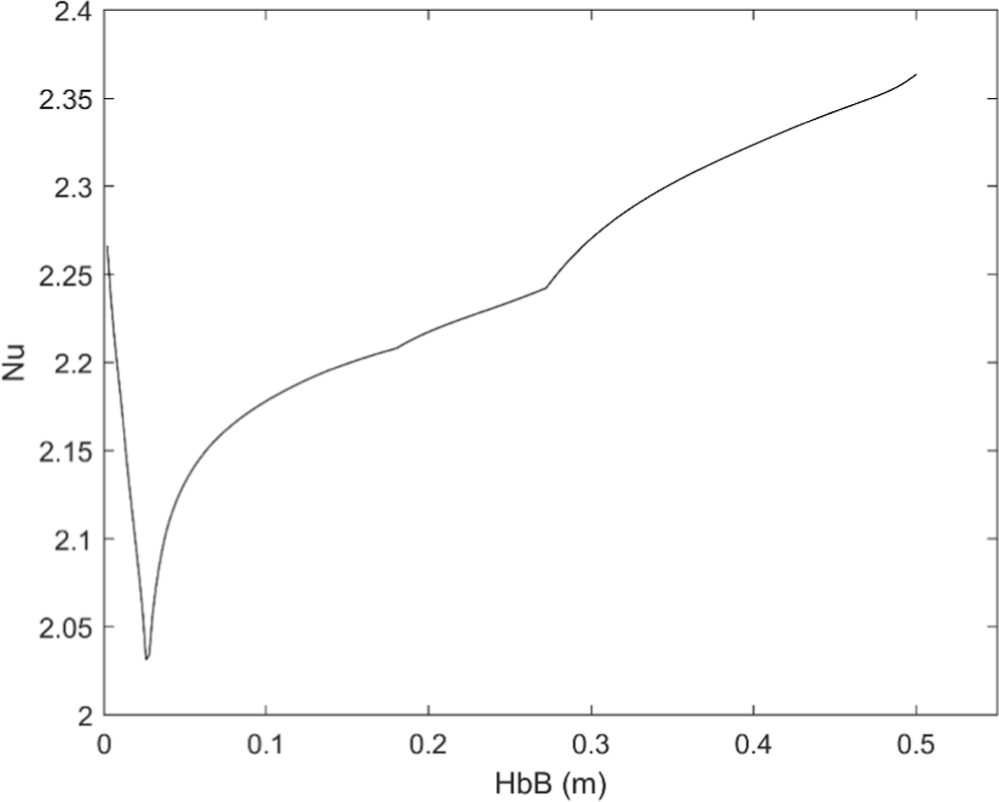
Nusselt number vs distance for a 72 μm particle in the reactor, case of 8% O_2_.

The main uncertainty regarding these calculations undoubtedly lies in the correction of the gas temperature profile near the injection tube (Section 5.2), which arises from the balance of a sufficient solid fuel feeding rate to provide enough sample in a reasonable time and the least possible affection to the flow prior to the particles’ injection. Figure 16 explores the effect of that correction on the calculated particle mass and temperature evolution in a (relatively) high oxygen concentration, more affected than in lower [O_2_] because of the respective length of the oxidation traces. As expected after Figure 10, the effect is notable on the particle maximal temperature, which increases in approximately 200 K; on the contrary, the curve OD vs distance is much less affected, with a rise in the oxidation rate equivalent to an increase of less than 1% in the oxygen concentration in the gas. The reason for this weak dependence is that, as indicated above, the apparent kinetics found adequate for the first oxidation stage is in fact nearly temperature-insensible, so the increase in particle temperature affects the oxidation rate essentially through the diffusivity, moreover through the mean of particle and gas temperatures. This is, in the authors’ opinion, an advantage of the mass-centered tests over the purely pyrometric measurements.

**Figure 16:**
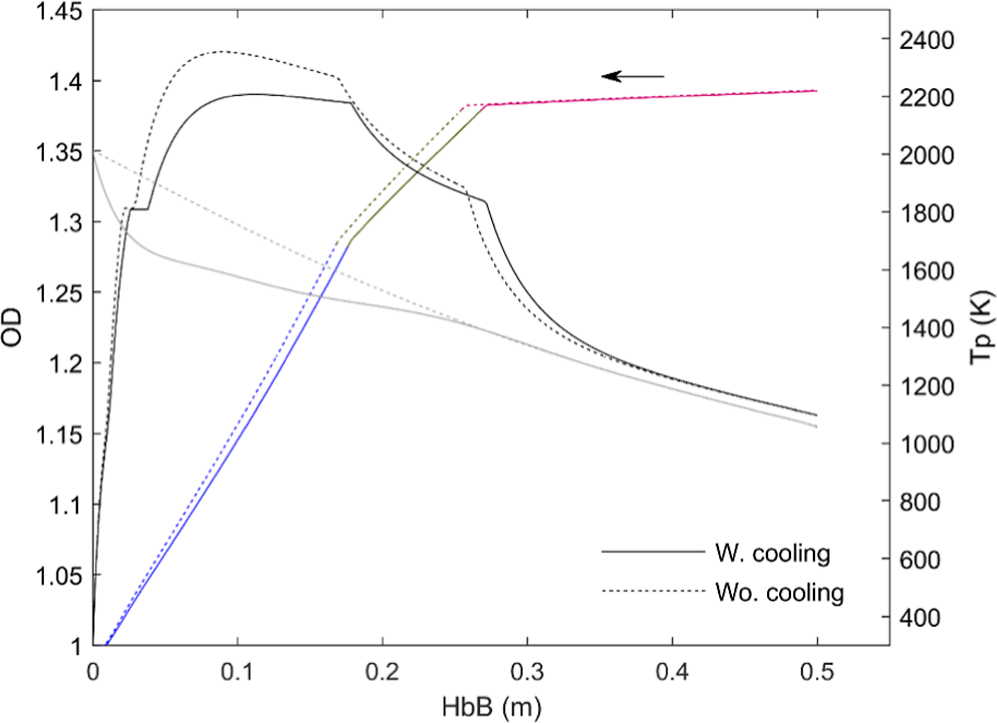
Effect of considering (continuous lines) or dismissing (dotted lines) the initial gas cooling caused by the particle stream. d_p_ = 72 μm, 12% O_2_.

### 5.5 Comparison with Previous Experiments with 75–90 μm Particles

As repeatedly mentioned through this text, in a previous work the authors studied the evolution of iron particles in a coarser size cut, namely 75–90 μm, including the corresponding oxidation curves, i.e. OD vs distance. Figure 17 compares those results with the predictions of the model with the kinetics shown in Table 2 and the corrected gas temperature profile. An average particle velocity profile was taken from Figure 5 in^14^ and a single particle diameter of 82 μm was used, determined as explained in Section 4 from the distributions in the reference quoted. The excellent agreement found in Figure 17 further extends the range of applicability of the model and supports the need for considering internal limitations for oxygen fixation in the burning particles in addition to its diffusion through the boundary layer.

**Figure 17:**
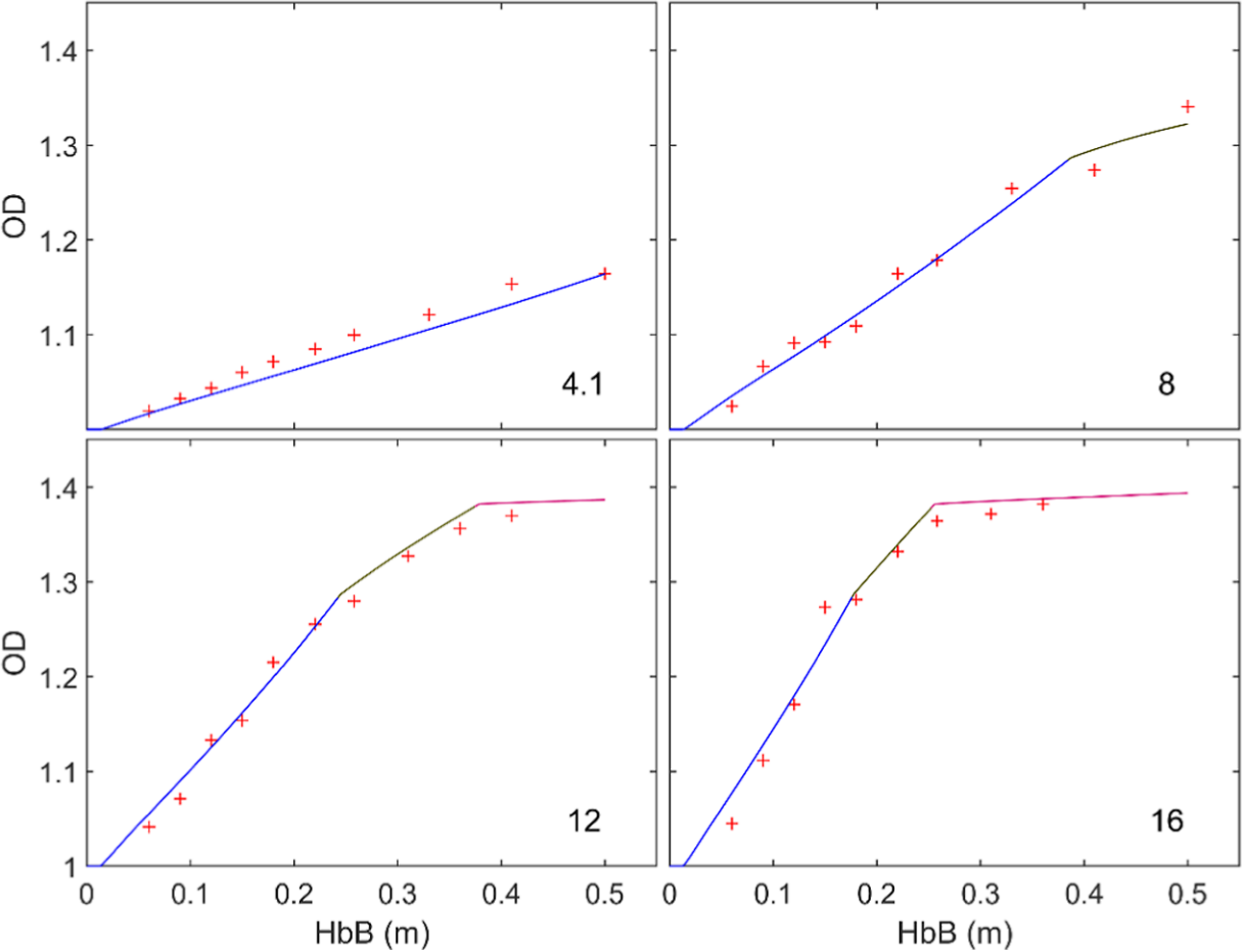
Oxidation degree vs distance between injection and sampling probe. Experiments with 75–90 μm [ref 14], calculations for an 82 μm particle with the kinetics in Table 2. The number in each subplot indicates the corresponding oxygen concentration in %.

## 6 Conclusions

The extension of a previous experimental study on direct iron powder oxidation in high temperature gases to smaller particles has confirmed the conclusions of that work, in particular the existence of consecutive oxidation stages, Fe → FeO → Fe_3_O_4_ → Fe_2_O_3_. The use of finer particles has provided a wider set of data regarding the evolution of the oxidation degree, especially in the range of highest Fe–O ratios. This data set has been used as a benchmark for comparison with a model which considers not only diffusion of oxygen through the particle’s boundary layer but also apparent kinetics as a means to take into account internal diffusion/transport and reaction.

External diffusion alone clearly overestimates the observed oxidation rates and the particle temperature profiles, which forces the introduction of other restrictions to oxygen incorporation into the particle. A single set of kinetic parameters (activation energy and pre-exponential factor) corrects most of the deviation, but is unable to reproduce the decrease in oxidation rate observed in the second and third stages. With three kinetics, an excellent agreement is found between predictions and experimental results in a wide range of conditions covering 4 to 16% O_2_ and two different size cuts, 63–75 and 75–90 μm (from a previous work). Whereas the uniformity of the particle’s behavior in the first two stages, leading to Fe_3_O_4_ in the samples, support a future attribution of the corresponding apparent kinetics to real processes (ion diffusion, convection in the droplet, chemical reaction, etc.), the kinetics given for Fe_3_O_4_ → Fe_2_O_3_ must be seen as statistically functional, since a diversity of phenomena are observed in this uppermost range of oxidation degrees in the reactor used, from extinction (which in fact is an extreme form of limitation to oxygen diffusion/fixation) to hyper-swelling.

The fit of the temperature profile shows an increasing overestimation of the expected maximal temperatures with oxygen concentration in the gas, which might be associated with metal evaporation, not considered in the model and beyond the scope of the present study. In order to fit the maximal particle temperature measured in an inert atmosphere, the gas temperature profile determined in the absence of particles had to be corrected to reproduce the cooling of the region close to the injection tube due to the stream of initially cold particles. The uncertainty associated with this correction, which is the largest in the simulations, has a noticeable effect in the prediction of the maximal particle temperatures, but affects only slightly the oxidation curves. In this sense, the general method is more robust in terms of oxidation curves (i.e., mass) than in what regards particle temperature profiles.

## Supplementary Material



## Notes

Additional graphs and photographs regarding different aspects of the experimental work (size distributions, iron particle traces, etc.) (PDF)
