# Supplementary material for: Apparent Kinetics for Direct Oxidation of Iron Particles Determined from Tests at High Temperature in a Flat Flame Reactor
Source: Energy Fuels. 2026 Mar 23;40(13):7171–81. doi: 10.1021/acs.energyfuels.6c00337 (PMC13073728; doi:10.1021/acs.energyfuels.6c00337)
Supplement: Supplementary file 1 [file ef6c00337_si_001.pdf]

Supporting Information for paper ef-2026-00337s

Apparent kinetics for direct oxidation of iron particles determined from tests at high temperature in a flat flame reactor

Santiago Jiménez [a,*], M. Carmen Mayoral [a], Luis M. Romeo [b]

[a] Instituto de Carboquímica-CSIC, Miguel Luesma 4, Zaragoza 50018, Spain.

[b] Aragon Institute of Engineering Research (I3A), Universidad de Zaragoza, Department of Mechanical Engineering, María de Luna 1, Zaragoza 50018, Spain.

* Corresponding author: yago@litec.csic.es, +34 976873616

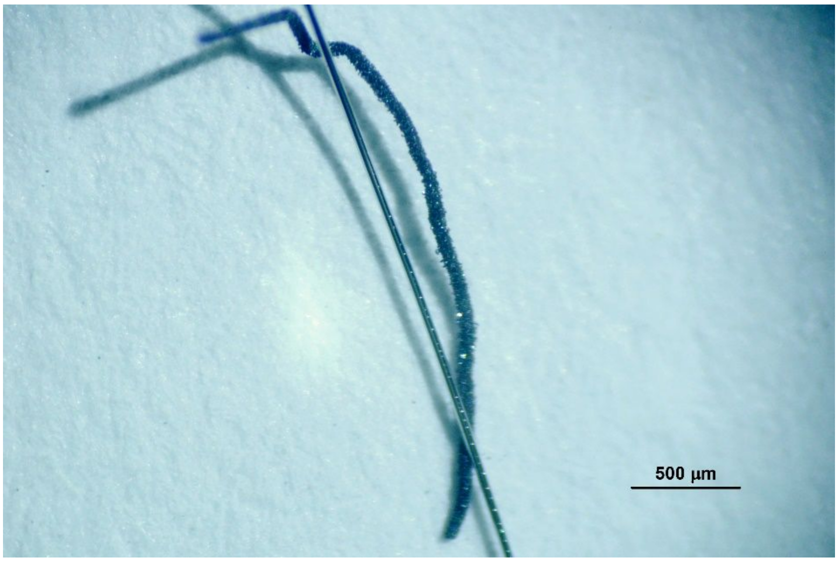

Figure SI1. Post-tests picture of a 75 µm platinum wire (straight, smooth) and a fragment of a ~112 µm iron oxide wire (originally 50 µm, pure iron)

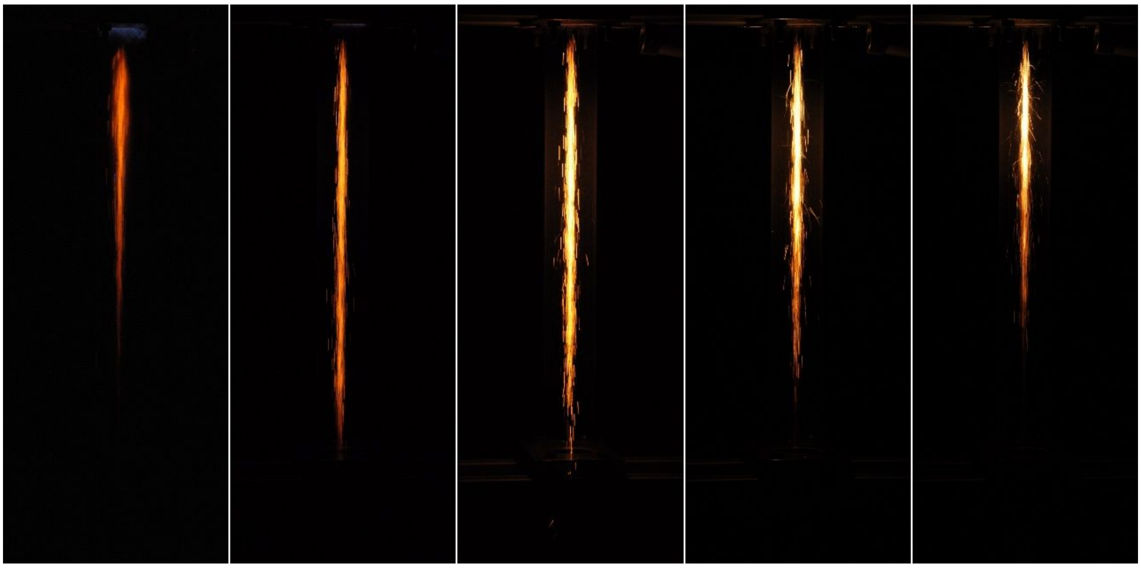

Figure SI2. General aspect of the iron ‘flames’. From left to right, 0.5, 4.1, 8, 12, 16% O_2. Time exposure 1/100 s. The tube length is 49 cm. The gas flat flame is hardly visible at top.

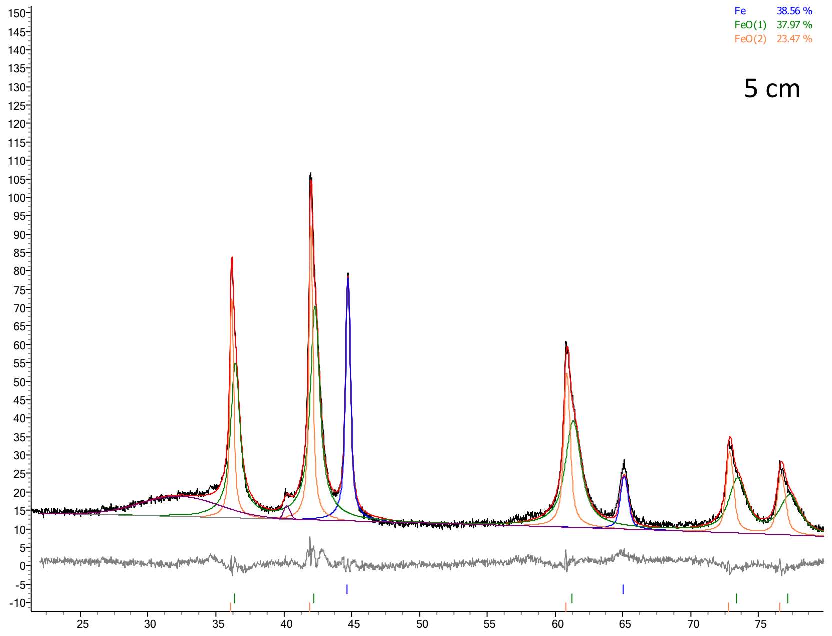

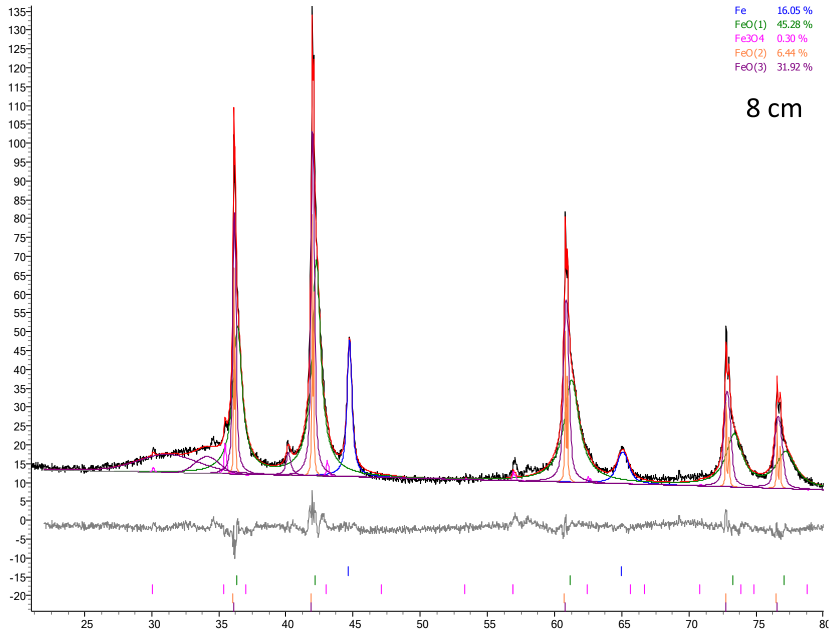

Figure SI3. X-ray diffractograms (black line) and Rietveld quantitative fit in samples collected at different heights below the burner (indicated below the legend). Note that FeO is represented by several compounds. (Continues)

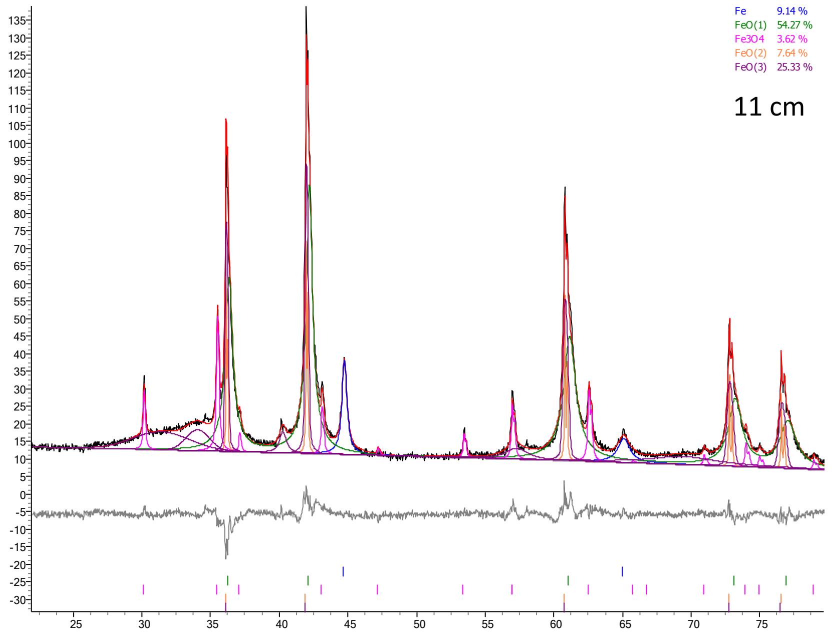

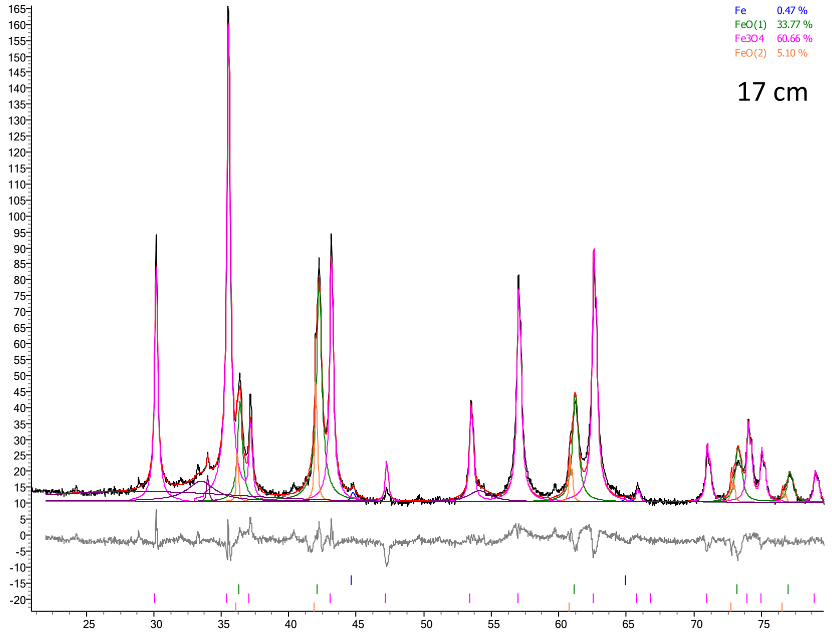

Figure SI3 (Continued). X-ray diffractograms (black line) and Rietveld quantitative fit in samples collected at different heights below the burner (indicated below the legend). Note that FeO is represented by several compounds. (Continues)

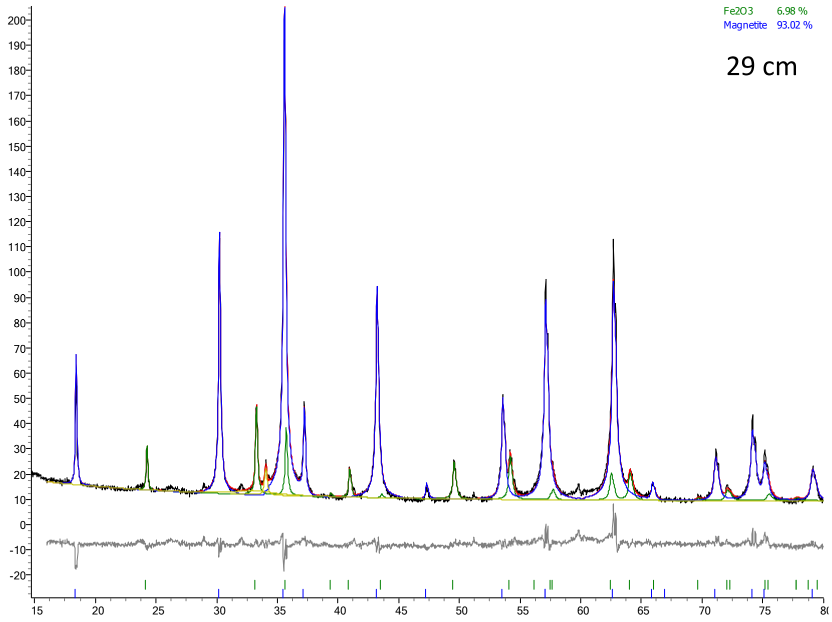

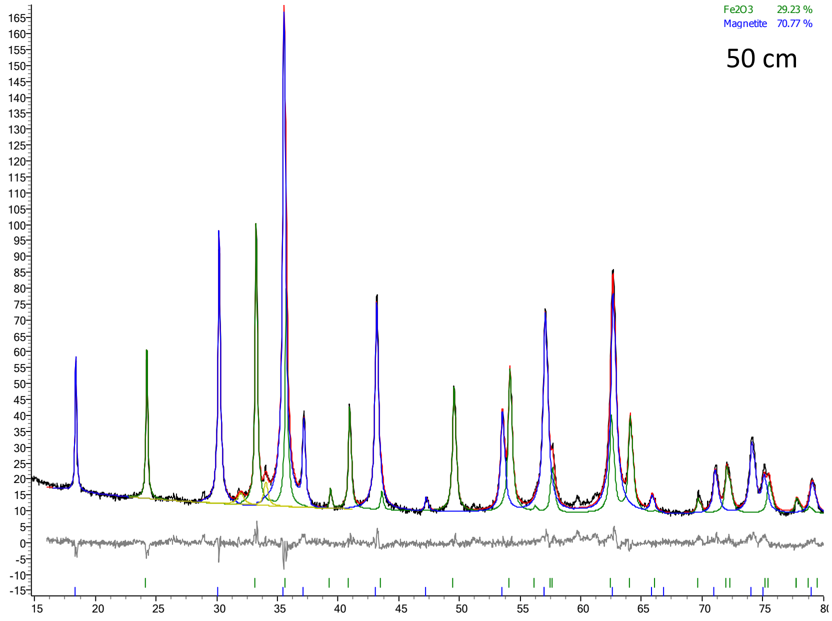

Figure SI3 (Continued). X-ray diffractograms (black line) and Rietveld quantitative fit in samples collected at different heights below the burner (indicated below the legend).

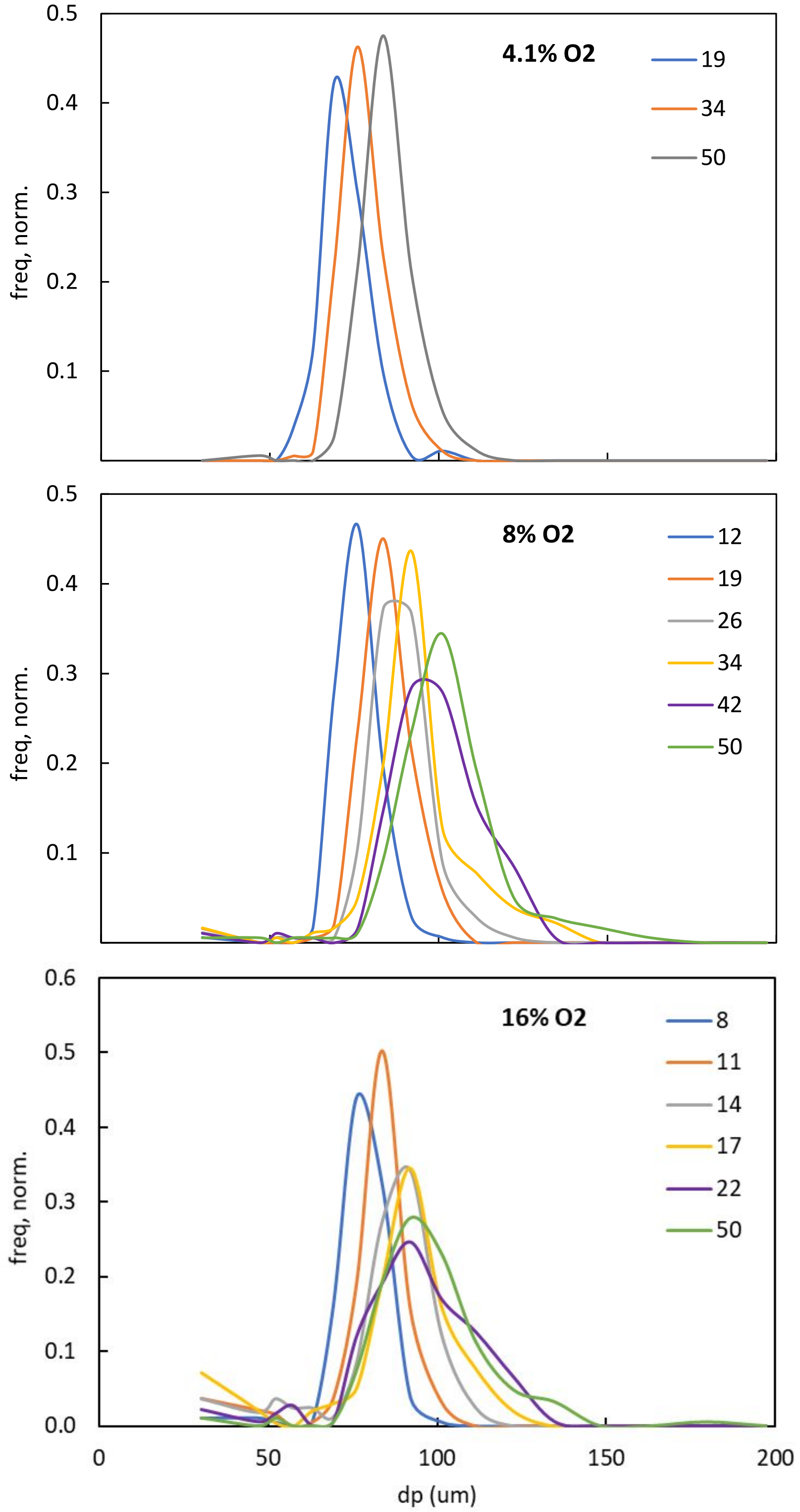

Figure SI4. Evolution of the particle size distribution (number-weighed) for the 63-75 μm cut in 4.1, 8 and 16% O_2. Legend shows HbB in cm.

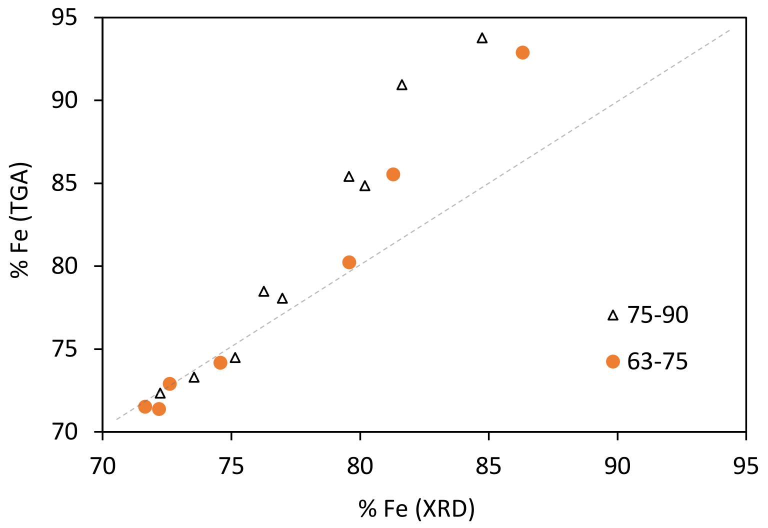

Figure SI5. Fraction of iron in the samples determined by TGA vs. XRD for the two size cuts studied in the present work and in a recent study with 75-90 μm particles.

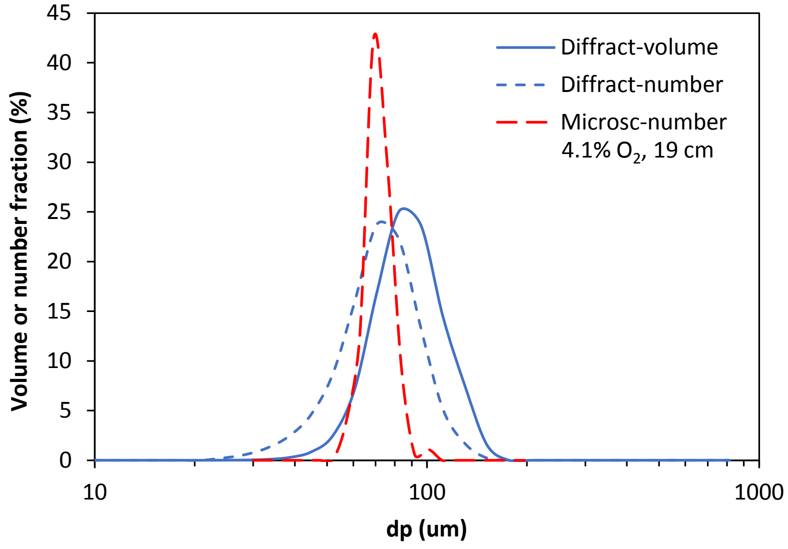

Figure SI6. Size distributions of the iron 63-75 μm particles. Blue, continuous: original fuel, laser diffractometer, volume-weighed (D_{43}~90 μm); blue, short-dashed: idem, but number-weighed (D_{10}~74 μm); red, long-dashed: number-weighed distribution in the sample with smallest oxidation degree but round aspect (D_{10}~72 μm).

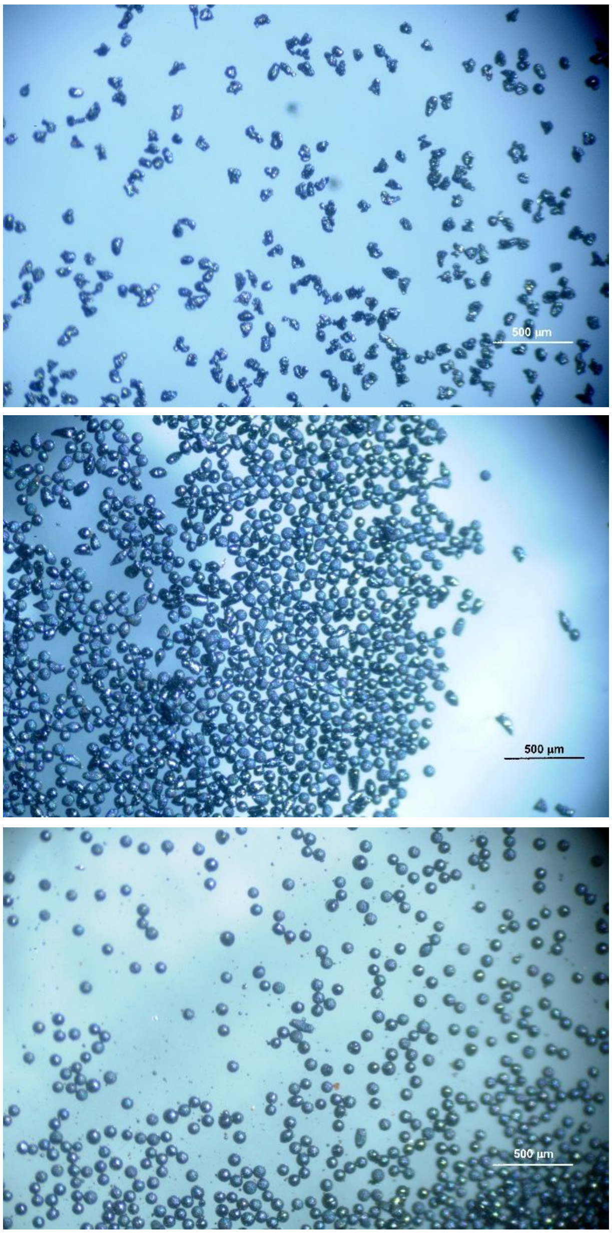

Figure SI7. Photographs in a microscope (x40) of the original 63-75 µm particles (top), and samples collected at 19 cm, 4.1% O_2 (center) and 12 cm, 8% O_2 (bottom).
